# Comprehensive profiling of paediatric chordoma: poorly differentiated and conventional subtypes

**DOI:** 10.1093/braincomms/fcag207

**Published:** 2026-06-03

**Authors:** Zejun Duan, Jing Feng, Haidan Wang, Junjie Yang, Zhong Ma, Song Han, Mingwang Zhu, Xiaolong Fan, Xueling Qi

**Affiliations:** Department of Pathology, Sanbo Brain Hospital, Capital Medical University, Beijing 100018, China; Department of Pathology, Sanbo Brain Hospital, Capital Medical University, Beijing 100018, China; Department of Neurology, Sanbo Brain Hospital, Capital Medical University, Beijing 100018, China; Department of Pathology, Sanbo Brain Hospital, Capital Medical University, Beijing 100018, China; Department of Pathology, Sanbo Brain Hospital, Capital Medical University, Beijing 100018, China; Department of Neurosurgery, Sanbo Brain Hospital, Capital Medical University, Beijing 100018, China; Department of Radiology, Sanbo Brain Hospital, Capital Medical University, Beijing 100018, China; Department of Pathology, Sanbo Brain Hospital, Capital Medical University, Beijing 100018, China; Department of Biology, Key Laboratory of Cell Proliferation and Regulation Biology, Ministry of Education, School of Life Sciences, Beijing Normal University, Beijing 100875, China; Department of Pathology, Sanbo Brain Hospital, Capital Medical University, Beijing 100018, China

**Keywords:** chordoma, poorly differentiated chordoma, SMARCB1 (INI1)-deficiency, DNA methylation profiling, genetic alteration

## Abstract

Chordoma in children is rare and inadequately characterized. This study aims to provide a comprehensive characterization of the clinicopathological and (epi)genetic features of chordomas in children, particularly the poorly differentiated chordomas (PDC). We established a cohort of seven PDCs and 28 conventional chordomas (CC). Besides characterizing the clinical and histopathological features, we performed comprehensive immunohistochemical staining for markers enabling diagnosis of chordoma and members of the sonic hedgehog signalling pathway. Using DNA methylation analysis, we characterized the molecular subtypes among chordomas in children and the SMARCB1 (INI1)-deficient tumours, which was further complemented by fluorescence in situ hybridization and targeted exome sequencing. Paediatric patients with PDC showed poorer prognosis compared with paediatric patients with CC. PDC tumours occurred more frequently in younger children and typically located at the clivus. Histologically, PDC tumours exhibited large polygonal/epithelioid cells or chubby spindle cells, with positive staining for cytokeratins and brachyury, but loss of expression of SMARCB1 (INI1) protein, and a high Ki-67 index. PDC samples showed promiscuous staining of sonic hedgehog pathway members, which contrasts the homogenous staining of sonic hedgehog pathway members in CC samples. Unsupervised hierarchical clustering and t-distributed stochastic neighbour embedding analyses of DNA methylation data showed that paediatric PDCs formed a distinct methylation cluster in comparison with the samples of CC, atypical teratoid/rhabdoid tumours and bone fide extracranial proximal epithelioid sarcoma. Whereas PDCs harboured relatively stable karyotype, frequent chromosomal gains or losses were observed in CCs. Mutational profiling identified different sets of genes in PDCs versus CCs. Loss of SMARCB1 (INI1) expression in PDCs was predominantly due to locus deletion. In summary, our findings support the differential diagnosis between paediatric PDC and CC. Further, our findings suggest that besides distinct methylome profiles, paediatric PDC and CC are likely driven by distinct pathogenic pathways.

## Teaser

Duan *et al*. characterized a cohort of paediatric chordomas. Compared to conventional chordomas, poorly differentiated chordomas were distinct in morphology, involvement of sonic hedgehog pathway and genomic alterations, and DNA methylation clusters. Dismal prognosis in patients with poorly differentiated chordoma was likely driven by *SMARCB1* loss and anatomic constraints.

## Graphical Abstract

**Graphical Abstract:**
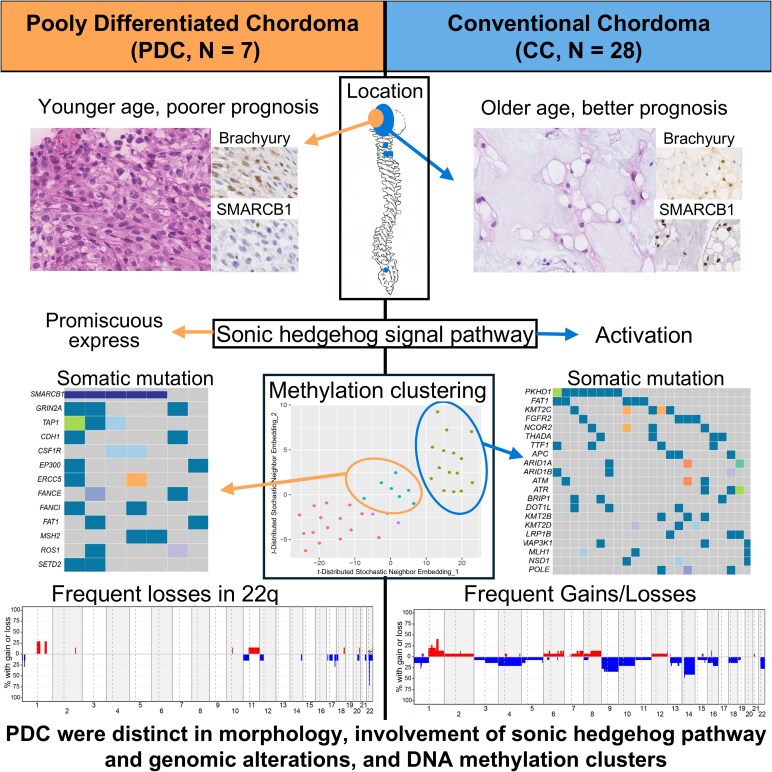

## Introduction

Chordomas are rare bone malignant neoplasms with notochordal differentiation, which mostly arise in the axial skeleton (including the skull base and the spine) with a high rate of local recurrence and metastasis.^1^ Chordomas may occur at any age with a peak incidence between 50 and 60 years of age, while less than 5% of cases occur before the age of 20 years.^2,3^ Approximately 12.5% skull base chordoma cases are diagnosed before the age of 20 years.^4^ The principal treatment is maximal safe surgical resection, which is often challenging owing to location related constrains and the destruction and infiltration of adjacent structures; consequently, rates of tumour residual and local recurrence are high.^5^ Chordomas are generally resistant to chemotherapy, however, adjuvant radiation therapy (RT) is recommended after surgery to postpone tumour progression and recurrence.^5-7^

Poorly differentiated chordoma (PDC) is a newly defined rare subtype of chordoma in the 2020 World Health Organization (WHO) ‘classification of tumours of bone’,^8^ which was previously recognized as ‘undifferentiated chordomas’, ‘cellular chordoma’, or ‘atypical chordoma’.^2^ Because chordomas with the loss of SWI/SNF-related matrix-associated actin-dependent regulator of chromatin subfamily B member 1 (SMARCB1 (INI1)) expression should be differentiated from malignant rhabdoid tumour (MRT), which also harbours loss of SMARCB1 (INI1),^9^ Hoch *et al*. proposed chordoma with SMARCB1 (INI1) loss as a new variant named PDC.^2^ Compared to conventional chordoma (CC), PDCs are more common in children and young adults, with a slight female predominance, and are characterized by the loss of SMARCB1 (INI1) expression.^7,10,11^ Deletions encompassing the *SMARCB1* locus have been suggested as a crucial pathogenetic mechanism in PDCs.^7^ Patients with PDCs have a more dismal prognosis compared with patients with CCs, which suggest that patients with PDCs should be treated aggressively with multimodality therapy.^2,12^

Sonic hedgehog (SHH) is a vital morphogen secreted by foetal notochord, responsible for guiding notochordal development. During the later stages of embryonic growth, SHH signalling becomes inactive, whereas continued activation of SHH signalling pathway may result in the transformation of notochordal cell rests into neoplastic cells.^13^ SHH and PTCH1 are expressed in human foetal notochord during early stages of development (between 12 and 28 weeks of gestation). SHH signalling pathway proteins, such as SHH, PTCH1, smoothened receptor (SMO) and glioma-associated homologue-1 (GLI1), are co-expressed or mutated in adult chordomas, and the extent of expression is correlated with prognosis.^13-15^ A preclinical model indicates that using SMO antagonist Vismodegib,^13^ SHH signalling pathway can be potentially target for chordoma treatment. Thus, aberrant activation of SHH signalling pathway appears to be involved in adult chordomas.^13,14,16^

Chordomas in children demonstrate a more aggressive behaviour compared to those in adults.^2^ Due to the rarity, there have been limited studies on chordomas in children, especially in PDCs.^2,17,18^ We previously conducted a comparison between PDCs and a group of SMARCB1 (INI1)-deficient tumours including primary adult sellar atypical teratoid/rhabdoid tumours (ATRTs), ATRTs in brain and bona fide proximal epithelioid sarcomas (PESs).^19^ Although we can confirm the diagnosis of PDC cases based on morphological and immunophenotypic characteristics, particularly the positive staining of brachyury and the loss of SMARCB1 (INI1) expression, the nature of this rare type of SMARCB1 (INI1)-deficient tumour remains inadequately characterized. In this study, we have collected a paediatric cohort comprising of seven PDCs and 28 CCs. Besides the clinical-pathological features, we further analysed the differences in the DNA methylation profiles and genetic alterations between paediatric PDCs and CCs.

## Materials and methods

### Tumour samples

Samples were retrieved from the pathological database of Sanbo Brain Hospital, Capital Medical University with the key word ‘chordoma’ for patients treated between March 2012 and April 2022. 351 cases from 272 patients were identified, 79 cases were from the operations at relapse. Among these cases, 48 (13.7%) were derived from 35 paediatric patients (18 years old or younger). Of the 35 primary paediatric chordomas, 28 (80%) were diagnosed as CC, and seven (20%) as PDC according to the 5th WHO classification of tumours of bone. Formalin fixed paraffin embedded (FFPE) samples from the primary operation were used in this study. Haematoxylin and eosin (H&E) stained sections were independently reviewed by two neuropathologists (Z.D. and X.Q.). Approval for conducting this study was obtained from institutional ethics committee at Sanbo Brain Hospital, Capital Medical University. Written informed consent was obtained from all patients and/or their legal representatives.

### Immunohistochemistry analysis

Immunohistochemical staining were performed on FFPE sections with the primary antibodies described below and the matching secondary antibodies. The immunohistochemical staining makers included: (i) cytokeratin markers and vimentin: pan-CK (Origene, AE1/AE3, 1:100 dilution), CK8 (Abcam, EP1628Y, 1:250 dilution), CK18 (Abcam, ab13326, 1:500 dilution), epithelial membrane antigen (EMA) (Dako, M29, 1:250 dilution) and vimentin (Abcam, EPR3776, 1:150 dilution); (ii) specificity and sensitivity marker for chordomas: brachyury (Abcam, EPR18113, 1:5000 dilution); (iii) SMARCB1 (INI1) (Santa Cruz Biotechnology, clone Y-7, 1:500 dilution) for evaluation of loss expression of SMARCB1 (INI1) protein; (iv) markers for tumours of glial, neural crest, or neuroendocrine origin: glial fibrillary acidic protein (GFAP) (Origene, EP13, 1:400 dilution), OLIG2 (Origene, EP112, 1:250 dilution), S-100 (Origene, 15E2E2 + 4C4.9, 1:100 dilution), synaptophysin (Syn) (Abcam, YE269, 1:200 dilution), chromogranin A (CgA) (Origene, LK2H10, 1:100 dilution) and CD56 (Origene, UMAB83, 1:200 dilution); (v) Ki-67 (Labvision, MIB-1, 1:50 dilution) for evaluation of tumour proliferation, P53 (Santa Cruz, sc-126, 1:200 dilution) for detection of P53 expression, with intense nuclear staining suggesting potential *TP53* mutation due to the accumulation of stabilized mutant protein.

The activation of SHH pathway was assessed by detecting SHH, PTCH1, SMO and GLI1 expression^14,15^ using the following antibodies: anti-SHH (Abcam, EP1190Y, 1:1000), anti-PTCH1 (Origene, OTI5C7, 1:200), anti-SMO (Origene, AP23648PU-N, 1:250) and anti-GLI1 (Bioss, bs-1206R, 1:200). The immunoreactive score (IRS) was used to evaluate the immunohistochemistry (IHC) data.^20^ The percentage of positively stained tumour cells was scored as: 0 (no positive cells), 1 (<10% positive cells), 2 (10–50% positive cells), 3 (51–80% positive cells), or 4 (> 80% positive cell). The intensity of staining was scored as: 0 (no expression), 1 (weak expression), 2 (moderate expression), or 3 (strong expression). The two scores were subsequently multiplied to generate IRS score: 0–1 (−, negative), 2–3 (1+, mild), 4–8 (2+, moderate), 9–12 (3+, strongly positive). The stainings were carried out using Leica Bond automated staining systems. The expression levels of the respective markers were independently assessed by two experienced pathologists, Z.D. and X.Q. In the case of disagreement, a consensus was achieved through collaborative discussion.

### Targeted exome sequencing

Next generation sequencing (NGS) was performed for seven paediatric PDCs and 28 CCs according to previously described protocol.^19^ FFPE samples were used for DNA extraction using the QIAamp DNA FFPE Tissue Kit (Qiagen). Sufficient DNA was obtained in 23 CC samples. Hybridization based target enrichment was carried out with GeneseeqOne™ pan-cancer gene panel (including 493 cancer relevant genes and MGMT, Geneseeq Technology Inc.) (Supplementary Table 1), and xGen lockdown hybridization and washing kit (Integrated DNA Technologies). The libraries were sequenced on the HiSeq4000 platform (Illumina) with 2 × 150 bp pair-end reads. Sequencing data were demultiplexed with bcl2fastq (v2.19), and analysed with Trimmomatic.^21^ The Genome Analysis Toolkit (GATK)^22^ was used to perform local realignments around indels and base quality reassurance. SNPs and indels were called with VarScan2^23^ and HaplotypeCaller UnifiedGenotyper in GATK, with the variant allele frequency (VAF) cut-off at 0.5% and a minimum of three unique mutant reads. To mitigate potential technical artefacts, we manually inspected variants in IGV and excluded those harbouring significant strand imbalance in read counts. Variant annotation was performed using VEP (Variant Effect Predictor). Germline variants were removed using the databases of dbSNP (https://www.ncbi.nlm.nih.gov/snp), 1000 Genome Projects (https://www.internationalgenome.org/data/), ExA (http://exac.broadinstitute.org/), ClinVar (https://www.clinicalgenome.org/data-sharing/clinvar/) and OMIM (https://omim.org/). TMB calculations were based on high-confidence variant data, which included only those mutations that have passed both artefact and germline filtering processes.

Sequence variants were classified for pathogenicity based on the standards and guidelines for the interpretation and reporting of sequence variants in cancer^24^: tier I (variants with strong clinical significance); tier II (variants with potential clinical significance); tier III (variants of unknown significance) and tier IV (benign or likely benign).

### Fluorescence in situ hybridization

Fluorescence in situ hybridization (FISH) analysis was performed on 4 μm FFPE sections using commercially available dual-colour probes for *SMARCB1* (located at 22q11) as the target and *EWSR1* (located at 22q12) as the control (Anbipin, Guangzhou, China). The sections were pre-treated in a 60°C oven for 30 min, deparaffinized in xylene/isopropanol and pressure cooked in a citrate buffer for 60 min. Following digestion in a coplin jar with 4 mg/ml pepsin at 37°C for 5–10 min, the sections were hybridized with the denatured probes at 37°C for 20 h. The slides were then washed and counterstained with DAPI. Hybridization signals were analysed with an Olympus fluorescence microscope equipped with imaging software. When there were more than 30% of tumour cells with 1 signal for the *SMARCB1* probe and 2 signals for the control probe, heterozygous deletion of *SMARCB1* was identified. When there were more than 30% of tumour cells with none signal for the *SMARCB*1 probe and 2 signals for the control probe, homozygous deletion of *SMARCB1* was identified.

### DNA methylation profiling

Using Illumina Infinium Human Methylation 850 (850k) BeadChip (Illumina, San Diego, USA), seven PDC and 28 CC samples were assessed for DNA methylation profiles. First, tumour DNA was extracted from FFPE samples using ReliaPrep FFPE gDNA Miniprep Kit (Promega, WI, Germany), and then restored using Illumina HD FFPE Restoration Kit (Illumina, CA, USA) according to the manufacturer’s instructions. Due to limitations in the quantity and quality of DNA preparations, only 15 CC samples were analysed for DNA methylation profiling. Second, DNA underwent bisulphite conversion, amplification, fragmentation and hybridization to the 850k BeadChip. Third, raw data were generated with the iScan array scanner and preprocessed using GenomeStudio software. Samples with a detection ratio of CpG sites greater than 95% (detection *P* value < 0.05) were used for subsequent analysis. Then, preprocessing and normalization were performed using the minfi package, beta and M values were calculated. Probes with the following three features were excluded from the analysis: (i) probes with detection *P* values > 0.01 (the detection *P* values were calculated as the total signal (M + U) for each probe to the background signal levels from the negative control probes.), (ii) probes on X or Y chromosome, (iii) probes containing SNPs (SNP 147) and (iv) probes mapping to multiple genome locations. Finally, 641 483 probes were kept for analysis. Unsupervised hierarchical clustering was performed using the 5000 most variably methylated probes across the dataset. Distance measure was 1-Pearson for the samples and euclidean for the CpG sites. Plots were generated with ggplot2. To perform unsupervised non-linear dimension reduction, the 5000 most variable probes were selected. The t-distributed stochastic neighbour embedding (*t*-SNE) plot was then computed via the R package Rtsne (version 0.15).

### Calibrated scores for individual samples

To analyse the DNA methylation profile in the context of sarcoma classifier,^25^ we reconstructed random forest (RF)-based sarcoma classification model by downloading the data of the sarcoma classifier reference cases from GSE140686, this enabled the generation of calibrated scores for individual samples. To construct the RF classifier model, the array preprocessing, batch effect removal and probe filtering criteria were the same as those described in the sarcoma classification model.^25^ During the construction, the RF algorithm (R package randomForest version 4.7-12) was used to generate 10 000 binary decision trees, The RF model was training with 10 000 CpGs with highest variable importance. The 3-fold cross-validation was performed. Each individual binary decision tree categorizes a given sample into one of the 65 classes, yielding aggregated raw scores. To facilitate comparisons of classifier performance across different classes, these raw scores are converted into the calibrated scores to reflect the confidence level of the class assignment. This transformation was implemented via an L2-penalized multinomial logistic regression calibration model, using the R package glmnet (version 4.1-8). Cross-validation of the RF classifier resulted in an estimated error rate of 1.58% for raw scores and 1.11% for calibrated scores and a multi-class area under receiver operating characteristic curve of 0.99 and a Brier score of 0.02. This indicates a high discriminating power. To obtain precise calibrated scores for chordoma samples, raw array data were preprocessed in strict accordance with the reference cases employed for the sarcoma classifier.

### Copy number variation analysis

Genome-wide DNA methylation array data were additionally analysed to evaluate copy number variations (CNVs) as described previously.^26^ The non-tumour brain tissues (NTB) (traumatic brain tissue resection) were used as the control. Using the ‘conumee’ R package (v.1.18.0)^27^ by the default parameters, the segment files were generated for individual samples with the raw intensity idat files. To obtain cumulated CNVs per group, the R package GenVisR (v.1.16.1)^28^ was used to process the segment files, which generated plots displaying the proportion of copy number losses/gains at the group level. The amplitude threshold was set at ± 0.15 to reduce the effects of noise.

### Statistical analysis

Data were analysed using SPSS statistics package software v24.0. Statistical significance was assessed using χ2 test, *t*-test, or Fisher's exact test. Overall survival (OS) was defined as the time from the date of diagnosis to chordoma-related death or the date of last visit. Kaplan–Meier curve, log-rank test and multivariable Cox analysis were used to assess survival difference. The *P* values of < 0.05 were considered statistically significant.

## Results

### Clinical features of paediatric PDCs

**Figure 1:**
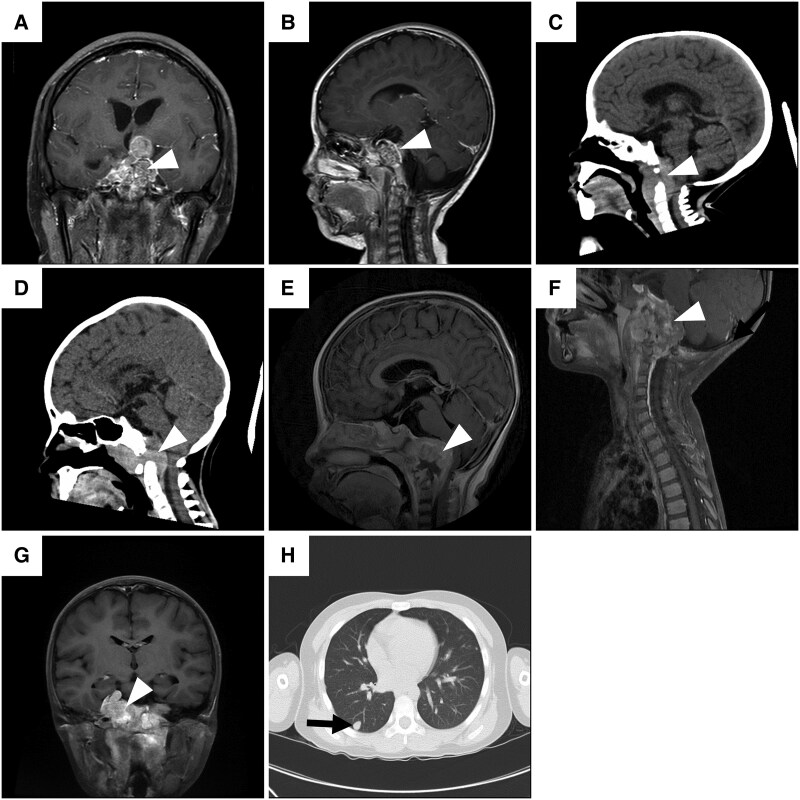
**Representative images of paediatric PDCs. Preoperative images of all seven paediatric PDCs are shown.** (**A**) (PDC_1), (**B**) (PDC_2), (**E**) (PDC_5), (**F**) (PDC_6) and (**G**) (PDC_7): tumours located at the clivus presented an uneven contrast enhancement on the post-contrast T1-weighted image (coronal or sagittal images). In PDC_1, PDC_5 and PDC_7, tumours also involved the sellar and suprasellar region (**A**), C1- C2 vertebrae (**E**) and right-side saddle (**G**), respectively. (**C**) (PDC_3) and (**D**) (PDC_4): sagittal CT images show an uneven patchy high-density mass at the clivus with bone resorption and destruction. The tumour sizes were 36 × 51 × 41 mm, 22 × 26 × 39 mm, 42 × 25 × 29 mm, 51 × 48 × 36 mm, 31 × 40 × 45 mm, 49 × 36 × 42 mm, and 58 × 44 × 45 mm for tumours shown in **A** to **G**, respectively. (**H**): PDC_7 with a metastasis in lung. Tumour regions are indicated with white triangles or black arrow. PDC, poorly differentiated chordoma; CT, computed tomography; mm, millimetre.

Paediatric PDCs were identified in seven patients (three males, four females), ranging in ages from 3 to 16 years (mean: 6.0 years, median: 3.0 years). All PDCs were located at the clivus, with three involving the sellar region, suprasellar region and sphenoid sinus, C1–C2 vertebrae and right-side saddle, respectively (Fig. 1 and Supplementary Table 2). All patients underwent surgical treatment, five of whom underwent craniotomy and two of whom underwent endoscopy. Two patients received adjuvant RT or proton therapy. Six patients succumbed to the disease, with OS ranging from 1 to 17 months. The remaining one patient (PDC_7) was alive with tumour metastasis to the spinal cord and lung at 22 months. The median overall survival time (OS) of patients with PDC was 3 months (Table 1).

**Table 1:** Comparison of clinicopathological features between paediatric PDCs and CCs

	PDC	CC	*P* value
Number	*N* = 7	*N* = 28	
Sex (female)	4 (57.1%)	15 (53.6%)	0.865
Age (mean and range, year)	6 (3–16)	11.1 (2–18)	**0**.019*
Location (skull base)	7 (100%)	25 (89.3%)	1
Histopathology			
Classic chordoma features	0	28 (100%)	
Mitoses (per 10 HPF, mean and range)	8.9 (4–14)	0.7 (0–4)	**0**.002*
Inflammatory cell infiltration	4 (57.1%)	12 (42.9%)	0.677
Invasion to surrounding tissues	6 (85.7%)	11 (39.3%)	**0**.041*
Necrosis	5 (71.4%)	10 (35.7%)	0.088
Immunophenotype			
Brachyury	7 (100%)	28 (100%)	
Epithelial markers (AE1/AE3, CK8, CK18, EMA)	7 (100%)	28 (100%)	
Loss of SMARCB1 (INI1) expression	7 (100%)	0	**＜0**.001*
S-100	3 (42.9%)	26 (92.9%)	**0**.002*
CD56	3 (42.9%)	28 (100%)	**＜0**.001*
Syn	6 (85.7%)	11 (39.3%)	0.076
Ki-67 index (mean, range)	38% (25–40%)	4% (1–10%)	**＜0**.001*
P53 over-expression	6 (85.7%)	1 (3.6%)	**＜0**.001*
Clinical outcome, OS (mo)			**＜0**.001*
Median survival time	Not reached	3 mos ^a^	
Five-year survival rate	75%	—	

PDC, poorly differentiated chordoma; CC, conventional chordoma; HPF, high power field; OS, overall survival; mo, month; *P < 0.05, Fisher’s exact test.

^a^The median progression-free survival was not reached.

The bold values in Table were significant statistical significance.

**Figure 2:**
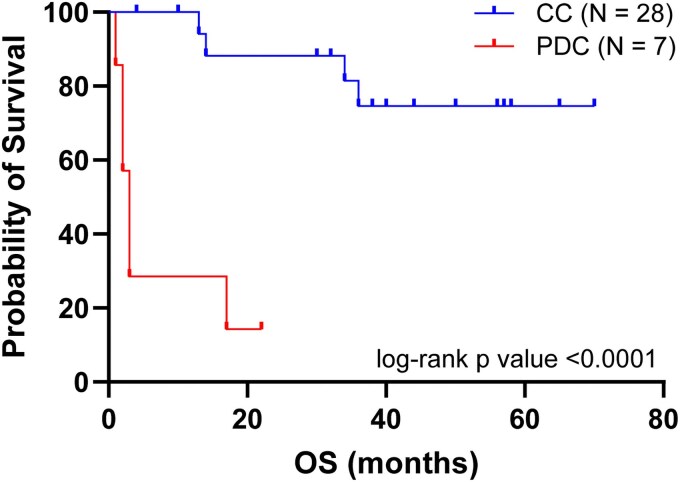
**Comparison of OS between paediatric patients with PDC and CC.** Kaplan–Meier plot with log-rank test of the OS data showed significantly worse prognosis in paediatric PDC patients compared to paediatric CC patients. OS, overall survival; PDC, poorly differentiated chordoma; CC, conventional chordoma.

Paediatric CCs were identified in 13 males and 15 females, their ages ranged from 2 to 18 years (mean: 11.1 years, median: 11 years). Twenty-five tumours were located at the skull base, including 24 at the clivus and one in the sellar region (Supplementary Fig. 1 and Supplementary Table 3). The remaining two were located in the cervical vertebrae and one in the sacral vertebrae. Twenty patients underwent craniotomy and eight underwent endoscopy. Seven patients received adjuvant RT, no patients received chemotherapy. Among the 21 patients with follow-up information, the OS ranged from 2 to 70 months and the five-year survival rate was 75% (Table 1 and Supplementary Table 3). The OS data show that paediatric patients with PDC had a significant poorer prognosis compared with paediatric patients with CC (log-rank test, *P* = <0.0001) (Fig. 2). Multivariable Cox analysis on the available clinical data indicates that while tumour type (PDC versus CC) was an independent prognostic factor, prognostic significance was not observed for other factors (age, tumour location, resection extent, and adjuvant RT) (Supplementary Table 4).

### Histopathological and immunophenotypic features of paediatric PDC

**Figure 3:**
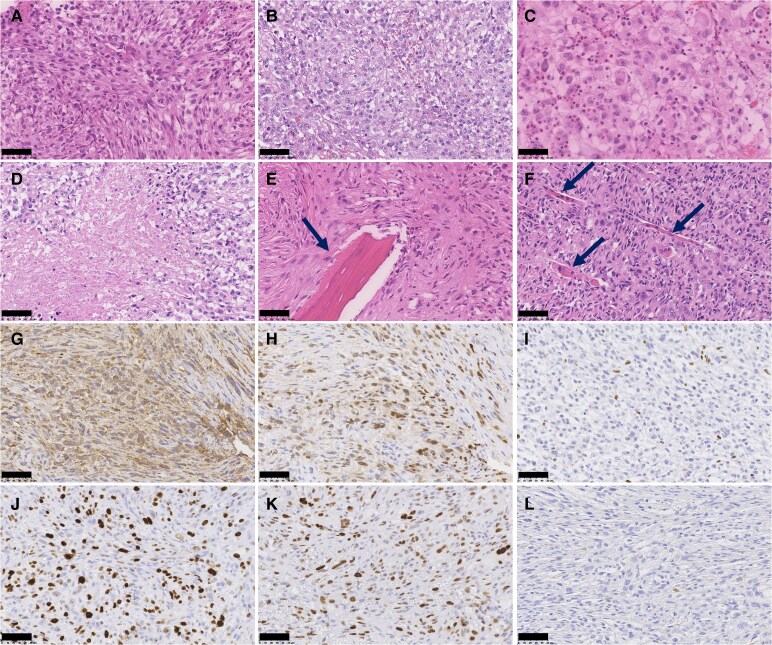
**Representative images of histopathology and immunophenotype of paediatric PDCs.** Images of chubby spindle tumour cells (**A**), large polygonal/epithelioid tumour cells (**B** and **C**), scattered neutrophils (**C**), focal necrosis (**D**), invasion to the surrounding bone (**E**) and striated muscle (**F**) are shown. In **E** and **F**, black arrows indicate the residual bone and striated muscle, respectively. PDCs showed diffuse positive AE1/AE3 staining (**G**), nuclear staining of brachyury (**H**), loss of SMARCB1 (INI1) expression in tumour cells, however SMARCB1 (INI1) staining was detected in vascular endothelial cells and inflammatory cells (**I**), Ki-67 staining in about 40% of the tumour cells (**J**), P53 over-expression (**K**) and negative S-100 staining (**L**). Scale bar: 50 μm. PDC, poorly differentiated chordoma.

Paediatric PDCs were characterized by cohesive solid sheets or nests of tumour cells, which included chubby spindle cells (*n* = 5), large polygonal/epithelioid cells (*n* = 1), or a combination of polygonal/epithelioid and spindle cells (*n* = 1) (Fig. 3A and B). The tumour cells typically exhibited round or oval nuclei with fine granular chromatin and visible nucleoli. Some nuclei appeared to be twisted and irregular. Mitotic figures were ranging from 4 to 14 per 10 high power field (HPF). Variable infiltration of inflammatory cells, with scattered or numerous neutrophils, was seen in four out of the seven cases (Fig. 3C). Focal or sheet necrosis was present in five of the seven cases (Fig. 3D). Invasion into surrounding tissues was noted in six of the seven cases (85.7%), including bone (*n* = 4), striated muscle (*n* = 2) and nerves (*n* = 1) (Fig. 3E and F). None of the seven cases exhibited a CC component.

In contrast, paediatric CCs displayed a lobulated architecture separated by fibrous septa (Supplemental Fig. 2A), comprised of nests and cords of tumour cells with abundant clear to eosinophilic or bubbly cytoplasm (physaliphorous cells) embedded in myxoid stroma (Supplemental Fig. 2B). Some cases exhibited solid sheets of epithelioid tumour cells, spindle cells and densely proliferative tumour cells (Supplemental Fig. 2C and D). The nuclei were generally small, with occasional nuclear atypia, pleomorphism, nuclear inclusions and multinucleated cells (Supplemental Fig. 2E). The mitotic activity was typically low or rare (fewer than 4/10 HPF). Focal or sheet necrosis was observed in 10 cases (35.7%), and invasion into bone tissues was identified in 11 cases (39.3%; Supplemental Fig. 2F). Lymphocytic infiltration was present in 12 cases (42.9%), occasionally accompanied by neutrophils and eosinophils.

All chordomas exhibited diffuse positive staining of epithelial markers (AE1/AE3, CK8, CK18 and EMA) and vimentin (Fig. 3G, Supplemental Figs 2G and 3). Diffuse nuclear expression of brachyury was observed in both PDCs and CCs (Fig. 3H and Supplemental Fig. 2H). PDC tumour cells were notably negative for SMARCB1 (INI1) protein expression (Fig. 3I), while CC samples retained SMARCB1 (INI1) expression (Supplemental Fig. 2I). The Ki-67 proliferation index ranged from 25% to 40% (mean: 38%) in PDCs (Fig. 3J), which is significantly higher than the Ki-67 proliferation index in CCs (Supplemental Fig. 2J), ranging from 1% to 10% (mean: 4%) (*t*-test*, P* < 0.05) (Table 1). Over-expression of P53 protein was observed in six PDCs (85.7%), but only in one CC (Fisher exact test, *P* < 0.05) (Fig. 3K, Supplemental Fig. 2K, and Table 1).

Interestingly, immunohistochemical analysis revealed consistent negativity for GFAP, OLIG2 and CgA in all cases. In contrast, S-100, CD56 and Syn showed variable positive staining in chordomas (Fig. 3L, Supplementary Figs 2L and 4). In consistency with previous reports,^1,12,29^ focal or partially weak expression of S-100 was observed in PDC samples, though significantly less intense compared to CC samples (Table 1). CD56 staining was positive in three PDC samples (42.9%) with focal or partial positivity in two samples, while nearly all CC samples exhibited diffuse positive staining (Fisher exact test, *P* < 0.001) (Table 1). Syn staining was positive in six PDCs (85.7%) versus 11 CCs (39.3%) (Fisher exact test, *P* = 0.076) (Table 1).

Collectively, these data demonstrate that while PDCs lack typical morphological features, they shared partial immunophenotypic overlap with CCs. Notably, PDCs were characterized by a high Ki-67 proliferation index, over-expression of P53 protein and loss of SMARCB1 (INI1) protein expression. Compared to CCs, paediatric PDCs showed significantly reduced S-100 and CD56 expression.

### Involvement of SHH pathway activation in paediatric PDCs and CCs

**Figure 4:**
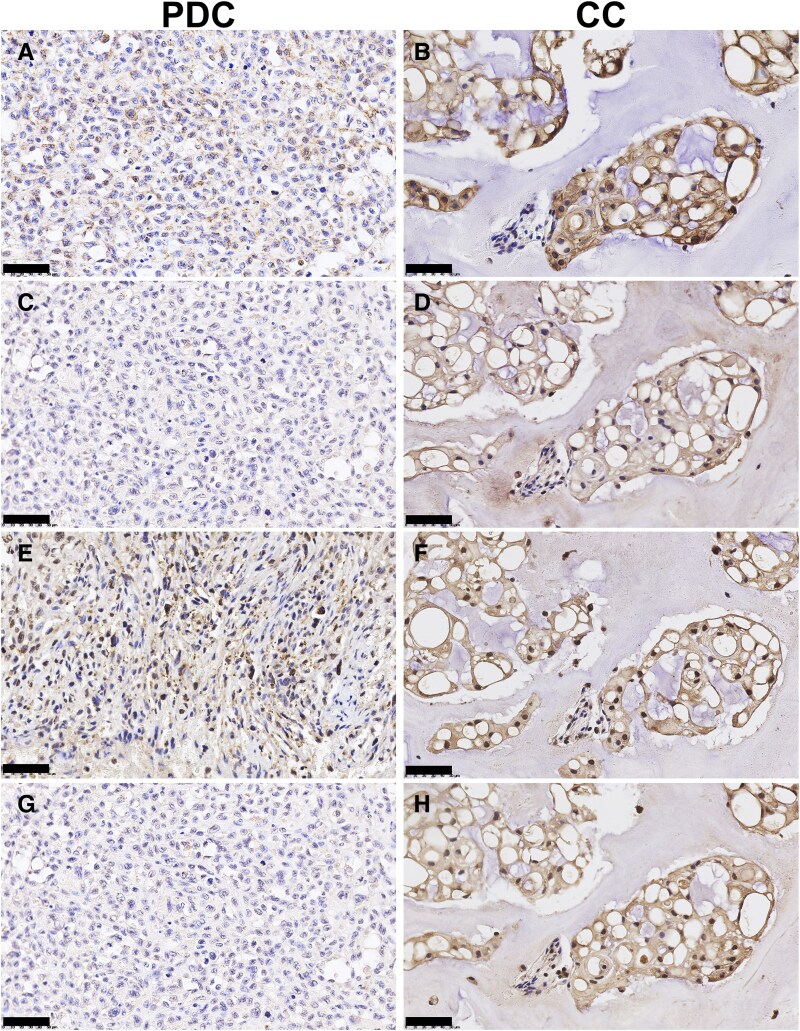
**Images of immunohistochemical stainings of SHH pathway members in representative PDC (left) and CC (right) samples.** Promiscuous staining pattern of SHH pathway members in a representative PDC sample (PDC_6, left) and homogenous staining pattern of SHH pathway members in a representative CC sample (CC_20, right) are shown. (**A**) and (**B**) were SHH staining, (**C**) and (**D**) were PTCH1 staining, (**E**) and (**F**) were SMO staining, (**G**) and (**H**) were GLI1 staining. Stainings were performed in consecutive sections, except for the SMO staining in PDC. Scale bar: 50 μm. PDC, poorly differentiated chordoma; CC, conventional chordoma.

While the SHH signalling pathway is normally active in the foetal notochord during early development, aberrant activation of this pathway has been reported in CCs.^13-15^ Here, we conducted immunohistochemical staining analyses for key SHH pathway members including SHH, SMO, PTCH1 and GLI1, on FFPE sections from seven PDCs and 25 CCs. IHC staining could not be performed for three CC samples due to insufficient tumour tissue availability. Staining results from consecutive sections were evaluated. Promiscuous staining patterns were observed in PDC samples; strong stainings of SHH and SMO were detected in four and three PDCs, respectively. Strong staining of GLI1 was detected in only one PDC, and strong staining of PTCH1 was not detected in PDC samples. In contrast, uniformly high level stainings for SHH, PTCH1, SMO and GLI1 were observed in most CC samples (Fig. 4 and Supplementary Table 5). Thus, SHH pathway activation likely differs between paediatric PDCs and CCs.

### Paediatric PDCs form a distinct methylation cluster

**Figure 5:**
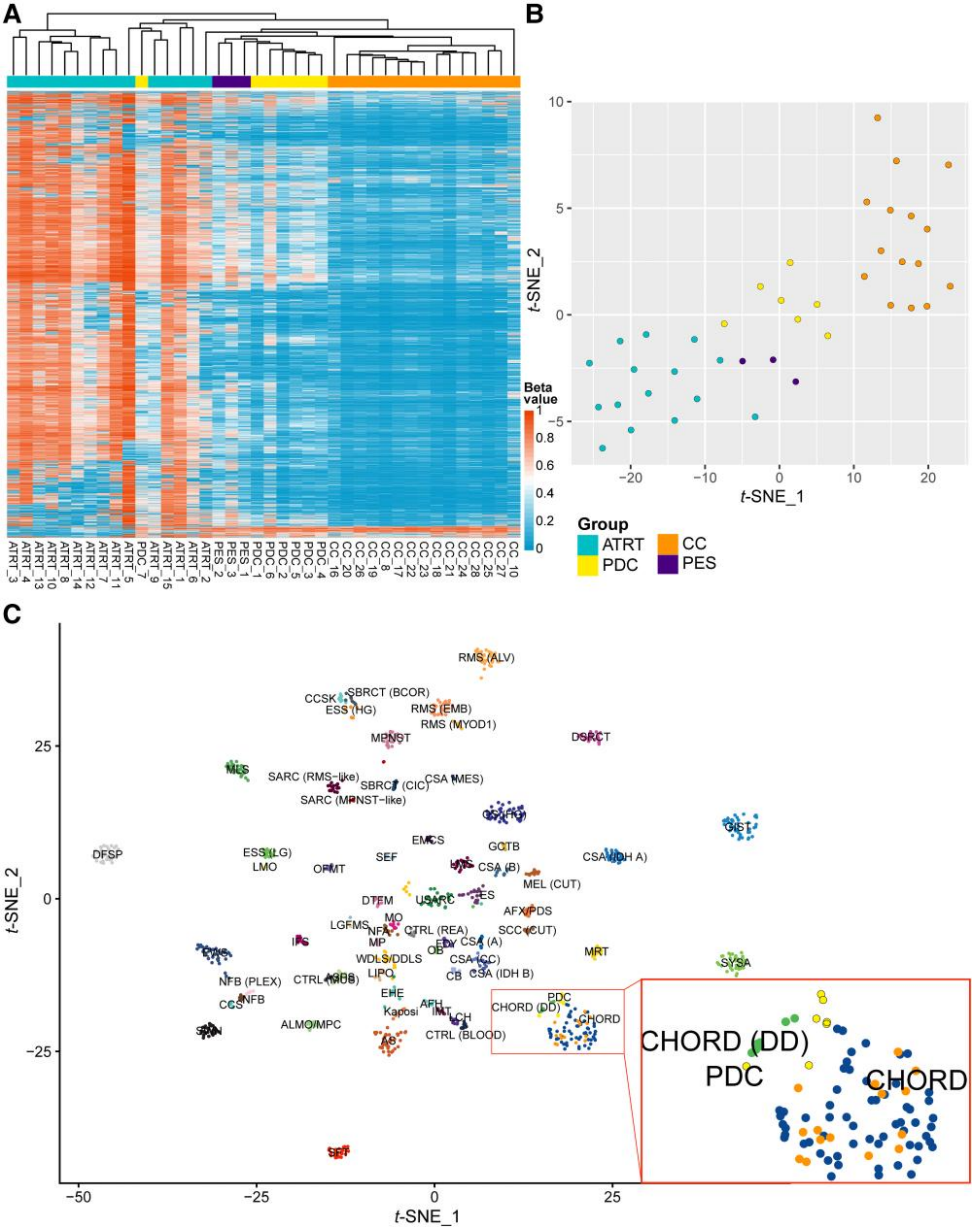
**Distinct DNA methylation profiles between paediatric PDCs and CCs.** Both unsupervised hierarchical clustering analysis (**A**) and *t*-SNE analysis (**B**) of our cohort show that paediatric PDCs (*N* = 7) exhibit a largely unique DNA methylation profile in comparison with CC (*N* = 15), ATRT (*N* = 15) and bone fide extracranial PES (*N* = 3) samples, however PDC_7 exhibited a methylation profile resembling ATRT samples. In **B**, each data point represents an individual sample, projected into 2D space via *t*-SNE using genome-wide DNA methylation beta-values, coloured by group: ATRT (teal), PDC (yellow), CC (orange), PES (purple). In the context of sarcoma classifier (**C**), CCs from our cohort perfectly overlapped with classical chordomas (labelled as CHORD) in GSE140686, whereas PDCs from our cohort closely clustered with the dedifferentiated chordomas (CHORD (DD)) in GSE140686, which are actually SMARCB1 (INI1)-deficient PDCs. Each data point represents an individual sample, projected via *t*-SNE using the sarcoma classifier’s predefined DNA methylation beta-values, labelled by disease/tumour type. The magnified region (lower right) details the sample identities, where each data point represents an individual sample: classical chordomas from GSE140686 (dark blue), dedifferentiated chordomas (GSE140686, green), PDCs from our cohort (yellow) and paediatric CCs (orange). PDC, poorly differentiated chordoma; CC, conventional chordoma; ATRT, atypical teratoid/rhabdoid tumour; PES, proximal epithelioid sarcoma.

With the available tumour materials, we conducted DNA methylation analysis on seven PDC and 15 CC samples. These data were further integrated with our previous data from SMARCB1 (INI1)-deficient tumours including three bone fide extracranial proximal epithelioid sarcomas (PES) and 15 ATRT samples.^19^ Both unsupervised hierarchical clustering and *t*-SNE analysis show that paediatric PDC, CC, PES and ATRT samples form distinct DNA methylation clusters (Fig. 5A and B).

Using *t*-SNE analysis, we further evaluated the DNA methylation profile of these tumours in the context of the reference cohort from the sarcoma classifier (GSE140686).^25^ CCs from our cohort perfectly overlapped with classical chordomas (CHORD) (Fig. 5C). Our PDCs clustered closely with the dedifferentiated chordomas (CHORD (DD)), which are actually SMARCB1 (INI1)-deficient PDCs in the sarcoma classifier.^25^ At the individual sample level, calibrated scores matching PDC to CHORD (DD) were > 0.99 for five PDCs or 0.77 for one PDC. However, the calibrated score for PDC_7 was 0.23. The details of calibrated scores and QC metrics for individual PDC samples are presented in Supplementary Table 6.

PDC_7 appeared to be an outlier. The results of hierarchical clustering analysis show that PDC_7 exhibited a methylation profile closely resembling ATRT samples. Compared with the other PDCs, the calibrated score for PDC_7 in individual subtyping with the sarcoma classifier was also low. However, the *t*-SNE plot shown in Fig. 5B indicates a considerable similarity between the DNA methylation profile of PDC_7 and other PDC samples. We also re-evaluated the clinical and pathological data for PDC_7. This tumour was located at clivus and right-side saddle (involving the dura matter and cranial nerve), and exhibited the typical morphological and immunohistochemical features of PDC, including diffuse expression of epithelial markers (AE1/AE3, CK8, CK18 and EMA) and brachyury, which are distinct from ATRTs. Thus, we classified this tumour as PDC, its atypical DNA methylation profile could be due to issues related to tissue quantities, tumour cell purity and quality of DNA preparation. Overall, our findings suggest that paediatric PDCs exhibit a largely unique DNA methylation profile, enabling differentiation between PDCs, CCs and other SMARCB1 (INI1)-deficient sarcomas (MRT, ES and bone fide extracranial PES) (Fig. 5C).

### Distinct patterns of genetic alterations between paediatric PDCs and CCs

**Figure 6:**
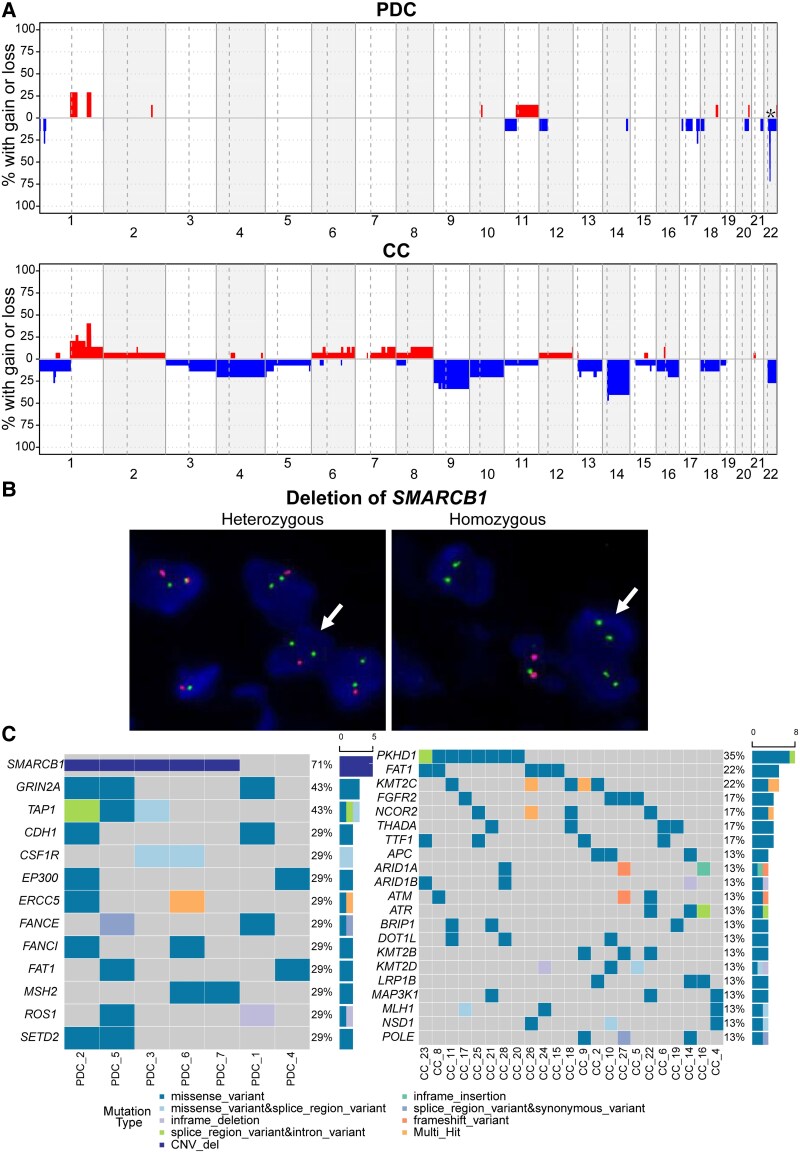
**Distinct patterns of genetic alterations in paediatric PDC and CC tumours examined.** (**A**) Chromosomal CNV profiles analysed using DNA methylation data. Paediatric PDC tumours (*N* = 7) exhibited a relatively stable chromosomal pattern, while CC tumours (*N* = 15) harboured complex gains or losses of chromosomal regions. Focal losses of *SMARCB1* at 22q in the PDC tumours are indicated with *. (**B**) Representative plot for *SMARCB1* deletion detected by FISH analysis in paediatric PDC (*N* = 7). Left: Image of heterozygous deletion of *SMARCB1* with one hybridization signal in magenta for the *SMARCB1* locus, but two signals in green for the control *EWSR1* locus in PDC_4. Right: Image of homozygous deletion of *SMARCB1* with no hybridization signal in magenta for the *SMARCB1* locus, but two signals in green for the control *EWSR1* locus in PDC_1. The white arrows indicate tumour cells with *SMARCB1* deletion. (**C**) Paediatric PDCs (*N* = 7) and CCs (*N* = 23) harbour distinct genetic alterations. Alterations found in at least two of the seven PDC (left) or three of the 23 CC (right) samples examined are shown. Each column represents an individual case, with alteration events denoted by gene (row) and type of the mutations (colour coded as per legend). In addition, overall frequency of each alteration is shown (colour coded based on mutation type). PDC, poorly differentiated chordoma; CC, conventional chordoma; CNV, copy number variation; FISH: fluorescence in situ hybridization.

Our genomic analysis revealed distinct patterns of genetic alterations between paediatric PDCs and CCs. DNA methylation array data was analysed to infer chromosomal copy number variation (CNVs). The results demonstrated that all seven paediatric PDC cases (100%) exhibited *SMARCB1* deletion at 22q (Supplementary Fig. 5), while no other recurrent CNVs were observed in this cohort (Fig. 6A). In contrast, CC samples exhibited a spectrum of recurrent CNV including *TBXT* amplification (6.7%, 1/15), *CDKN2A/B* deletion (33.3%, 5/15), *PTEN* deletion (13.3%, 2/15), as well as recurrent losses at chromosome 14q (40%, 6/15), 22q (20%, 3/15). *SMARCB1* copy number loss was detected in 33.3% (5/15) of CCs by methylation array. However, low *SMARCB1* copy number signals in CC samples could be caused by technique limitations of DNA methylation array and/or tumour purity, as all CCs retained SMARCB1 (INI1) protein expression as detected by IHC analysis.

FISH analysis confirmed *SMARCB1* deletions in all PDC cases (six heterozygous, one homozygous) (Fig. 6B). In all seven PDC cases, two signals were consistent detected with the control probe for *EWSR1*, indicating that *EWSR1* was not deleted or re-arranged. Targeted exome sequencing revealed heterozygous *SMARCB1* copy number deletions (mean CNV: 1.1) in five of the seven PDCs. The observed discordance between bulk DNA assays (methylation array, exome sequencing) and FISH analyses may primarily stem from tumour purity effects, as admixed non-neoplastic cells can mask homozygous deletions as heterozygous calls in bulk analyses.

Results of targeted exome sequencing showed PDCs harboured recurrent alterations in *GRIN2A*, *TAP1*, *CDH1*, chromatin remodellers (*EP300* and *SETD2*) and genome stability regulators (*ERCC5*, *FANCE*, *FANCI* and *MSH2*) (Fig. 6C and Supplementary Fig. 6). CCs displayed mutations in a distinct set of genes, including *PKHD1* (34.8%, 8/23), histone methyltransferases (*ARID1A/B*, *KMT2B/C/D*, *DOT1L* and *NSD1*), DNA damage response genes (*ATM*, *ATR* and *BRIP1*) and transcriptional regulators (*NCOR2* and *TF1*). Despite frequent P53 over-expression, *TP53* mutations were rare (one PDC case, c.472C > G p.R158G missense mutation, VAF: 18.58%). The majority of recurrent variants in both cohorts were classified as tier III. Both cohorts showed low tumour mutational burden (PDCs: mean 1.7 mut/Mb; CCs: mean 1.9 mut/Mb), mutations in MGMT promoter were not detected.

Our results validate and extend the studies of Owosho *et al*.^30^ demonstrating that *SMARCB1* copy number loss constitutes the most prevalent genomic alteration in paediatric PDCs. In addition, we show that paediatric PDCs displayed a relatively stable genome compared to CCs, while CCs commonly harboured CNVs in *CDKN2A/B*, *PTEN*, *TBXT* and in chromosomal regions such as 14q and 22q. Further, heterozygous deletion of *SMARCB1* was more common than homozygous deletion in paediatric PDCs.^31^ Thus, paediatric PDC and CC cases exhibited distinct recurrent genomic alterations.

## Discussion

Here, we report the characterization of a paediatric cohort of seven PDCs and 28 CCs. Paediatric PDCs were typically located at the clivus, occurred in younger children, and their prognosis was significantly poorer compared with children with CC. Unlike the uniform strong expression of SHH pathway members in paediatric CCs, SHH pathway members showed promiscuous expression in PDCs. PDCs and CCs formed distinct DNA methylation clusters. While PDCs harboured stable karyotype with the loss of the *SMARCB1* region as a predominant feature, CCs harboured complex chromosomal copy number abnormalities. Though a subset of recurrent mutations in PDCs or CCs are involved in overlapping pathways (e.g. chromatin remodelling), other recurrent mutations are involved in distinct pathways. Overall, our findings reinforce the differential diagnosis between PDCs and CCs, and extend previously reported distinctions between PDCs and CCs in that PDCs and CCs are likely driven by different pathogenic pathways.^32^

Despite the limited number of cases, to our knowledge, this is the first attempt to detect the expression of SHH pathway members in paediatric PDC samples. Though promiscuous co-expression of SHH pathway members was observed in PDC samples, their expression was remarkedly weaker compared with the homogenous and strong expression in the CC samples. Our findings support the notion that SHH signalling activities are differentially involved in paediatric PDC and CC, though additional gene expression analyses are required to confirm these findings.

PDC is a rare type of SMARCB1 (INI1)-deficient tumours, characterized by its distinct clinicopathological features and specific DNA methylation profile.^7,12,33^ PDCs in the paediatric age group need to be differentiated from other SMARCB1 (INI1)-deficient tumours such as ATRT, MRT and PES. Sande *et al*.^34^ have reported that PDCs with *SMARCB1* loss and located in the knee joint are aggressive malignancies, and their DNA methylation profiling shows that the four cases cluster with axial PDC. In our report, although only a limited number of SMARCB1 (INI1)-deficient tumours have been tested, our findings confirm that paediatric PDCs form a methylation cluster independent from other SMARCB1 (INI1)-deficient tumours (e.g. ATRT and bona fide PES). In our cohort, PDC_7 had a methylation profile similar to ATRT samples in unsupervised hierarchical clustering. However, the clinical, morphological and immunophenotypic features of this tumour were distinct from ATRTs. Aldape *et al*.^35^ emphasized that while DNA methylation is a useful diagnostic tool, the results should be interpreted within the entire context of a given case. Thus, we confirmed that this tumour was PDC, not ATRT. The observation may reflect limitations of our study cohort size as well as the inherent technical variability in the DNA methylation profile-based classification of central nervous system (CNS) tumours.

SMARCB1 (INI1)-deficient tumours include various types of CNS tumours and sarcomas, such as ATRT, cribriform neuroepithelial tumour, MRT, epithelioid sarcoma, epithelioid malignant peripheral nerve sheath tumour, and myoepithelial carcinoma.^9,36,37^ Loss of SMARCB1 (INI1) expression is highly correlated with *SMARCB1* inactivation. Consistent with previous reports of heterozygous or homozygous deletion at the *SMARCB1* locus,^7,33^ our results from DNA methylation profiling, FISH analysis and targeted exome sequencing together showed that at least five of the seven PDCs harboured deletion of the *SMARCB1* region. Though previous studies have reported duplications of *TBXT* in 27% of adult chordomas,^38,39^ no amplification or gain of *TBXT* was observed in the PDCs of our cohort. In contrast, *TBXT* amplification was detected in 6.7% of our CC samples. Loss of *CDKN2A/2B* and *PTEN* were also only detected in CC samples of our cohort, at rates of 33.3 and 13.3%, respectively. Unlike the complex chromosomal copy number abnormalities in CCs, PDCs exhibit relatively stable karyotypes.^32^ These results reinforce the findings of other reported cohorts that deletions of the *SMARCB1* region, leading to the loss of SMARCB1 (INI1) expression, likely account for a crucial pathogenetic mechanism in PDCs.^7,10,11,32^

Besides copy number abnormalities, numerous genetic events have been observed in adult CCs, including activating mutations in PI3K (including *PIK3CA*, *PIK3R1* and *PTEN*) and mTOR pathway signalling genes,^1,38,39^ mutations in chromatin remodelling genes (such as *ARID1A*, *PBRM1* and *SETD2*),^4,14,39,40^ mutation in *TERT* promoter regions,^40^ and inactivating mutation in *LYST.*^38,39^ Our findings confirmed that paediatric PDCs and CCs are likely driven by distinct pathogenic pathways. First, apart from *SMARCB1* loss, recurrent genetic alterations observed in our paediatric cohort differed from the previously reported genetic alterations in adult CCs.^38,41^ Second, recurrent mutant genes in paediatric PDCs and CCs are involved in distinct pathways. Though dysregulated chromatin remodelling is involved in both subsets of paediatric chordomas, PDCs are predominantly affected by the loss of *SMARCB1*, whereas CCs are affected by mutations in a set of histone methyltransferases such as *SETD2*. Therefore, our results support the notion that PDC represents a distinct type of tumour that is genetically different from CC.^32^

PDCs are aggressive but have comparably stable genome. The following aspects could play causative roles. In line with the clinical behaviour of malignant rhabdoid tumours,^42^ despite low rate of gene mutation and CNV, *SMARCB1* loss may play a pivotal role in PDC pathogenesis. Further, anatomical complexity in the skull base and consequences of aggressive resection may have also impeded the survival outcome.

Our study stemmed from its single-centre design and the relatively small cohort size (*n* = 35), which may constrain the statistical power of our findings. Further, RNA sequencing was not conducted as fresh samples were not available. Given the inherent limitations of FFPE samples, mRNA molecules are frequently degraded and chemically modified, we opted not to perform FFPE RNA sequencing. Consequently, we only carried out staining of SHH pathway members on FFPE samples. Further, reliance on immunohistochemical staining alone to evaluate the activity of the SHH signalling pathway could potentially compromise the accuracy of our findings. Third, our targeted exome sequencing panel did not include *TBXT* and *LYST*, both of which are known to be frequently altered in chordoma. Finally, *EWSR1* was used as the control for the detection of *SMARCB1* loss, which may be suboptimal. Because large deletions affecting the *SMARCB1* locus may also involve the *EWSR1* locus, which could complicate the interpretation of FISH results.

In summary, paediatric PDC exhibits unique morphological, immunophenotypic and (epi)genetic features. Due to the dismal prognosis, patients with PDC require aggressive treatment involving multiple modalities. Improved differential diagnosis and genetic characterization may facilitate the development of new treatment strategies for young children with chordoma.

## Supplementary Material

fcag207_Supplementary_Data

## Data Availability

The data supporting the findings of this study are available on request from the corresponding author, Prof. Xueling Qi. The code used to perform the analysis is available through a repository at https://github.com/InkLeafNotes/RF_classifier.
