# Supplementary material for: Comprehensive profiling of paediatric chordoma: poorly differentiated and conventional subtypes
Source: Brain Commun. 2026 Jun 3;8(3):fcag207. doi: 10.1093/braincomms/fcag207 (PMC13263051; doi:10.1093/braincomms/fcag207)
Supplement: fcag207_Supplementary_Data [file fcag207_supplementary_data.pdf]

Supplementary Table 1. List of 493 cancer-related genes

ABCB1(MDR1)	ABCC2(MRP2)	ACVR1	ADGRB3 (BAI3)	ADH1B	AFDN (MLLT4)
AIP	AKT1	AKT2	AKT3	ALDH2	ALK
AMER1 (FAM123B)	APC	AR	ARAF	ARID1A	ARID1B
ARID2	ARID5B	ASCL4	ASXL1	ATF1	ATIC
ATM	ATR	ATRX	AURKA	AURKB	AXIN2
AXL	B2M	BAD	BAK1	BAP1	BARD1
BAX	BCL2	BCL2L11 (BIM)	BCOR	BCORL1	BCR
BIRC3	BLM	BMPR1A	BRAF	BRCA1	BRCA2
BRD4	BRIP1	BTG2	BTK	BUB1B	CASP8
CBL	CBLB	CCDC26	CCN6 (WISP3)	CCND1	CCND2
CCND3	CCNE1	CD274 (PD-L1)	CD74	CDA	CDC73
CDH1	CDK10	CDK12	CDK4	CDK6	CDK8
CDKN1A	CDKN1B	CDKN1C	CDKN2A	CDKN2B	CDKN2B-AS1
CDKN2C	CEBPA	CEP57	CHD4	CHD7	CHD8
CHEK1	CHEK2	CIC	CREBBP	CRKL	CSF1R
CSNK2B	CTCF	CTDNEP1	CTLA4	CTNNB1	CUL3
CUX1	CXCR4	CXXC5	CYLD	CYP19A1	CYP2A13
CYP2A6	CYP2A7	CYP2B6	CYP2C19	CYP2C9	CYP2D6
CYP3A4	CYP3A5	CYSLTR2	DAXX	DDR2	DDX31

DDX3X	DENND1A	DHFR	DICER1	DKK1	DLL3
DNMT3A	DOT1L	DPYD	DTL(CDT2)	DUSP2	EGFR
EIF1AX	EML4	EMSY(c11orf30)	EP300	EPAS1	EPCAM
EPHA2	EPHA3	EPHA5	ERBB2 (HER2)	ERBB3	ERBB4
ERBIN (ERBB2IP)	ERCC1	ERCC2	ERCC3	ERCC4	ERCC5
ESR1	ETV1	ETV4	ETV5	ETV6	EWSR1
EXT1	EXT2	EZH2	EZR	FAM131B	FANCA
FANCC	FANCD2	FANCE	FANCF	FANCG	FANCI
FANCL	FANCM	FAT1	FBXW7	FGF19	FGFR1
FGFR2	FGFR3	FGFR4	FH	FLCN	FLT1 (VEGFR1)
FLT3	FLT4 (VEGFR3)	FOXA1	FOXL2	FOXO1	FOXP1
FOXR2	FRG1	FUBP1	GABRG1	GATA1	GATA2
GATA3	GATA4	GATA6	GFAP	GFI1	GFI1B
GLI1	GLI2	GNA11	GNAQ	GNAS	GPS2
GREB1	GREM1	GRIN2A	GRM3	GRM8	GSE1
GSTM1	GSTM4	GSTP1	GSTT1	H3-3A (H3F3A)	HDAC2
HDAC9	HGF	H3C2 (HIST1H3B)	H3C3 (HIST1H3C)	HLA-A	HNF1A
HNF1B	HOXB13	HRAS	IDH1	IDH2	IFNA6
IFNB1	IFNE	IFNG	IFNGR1	IFNGR2	IGF1R

IGF2	IKBKE	IKZF1	IL7R	INPP4B	IRF2
JAK1	JAK2	JAK3	JARID2	JUN	KBTBD4
KDM4C	KDM5A	KDM6A	KDR (VEGFR2)	KEAP1	KIAA1549
KIF1B	KIT	KITLG	KLLN	KMT2A (MLL)	KMT2B
KMT2C (MLL3)	KMT2D (MLL2)	KRAS	LDB1	LHCGR	LMO1
LRP1B	LYN	LZTR1	MAP2K1 (MEK1)	MAP2K2 (MEK2)	MAP2K4
MAP3K1	MAP3K4	MAX	MC1R	MCL1	MDM2
MDM4	MECOM	MED12	MEF2B	MEN1	MET
MGMT	MITF	MLH1	MLH3	MLLT1	MLLT3
MN1	MPL	MRE11(MRE11A)	MSH2	MSH6	MTAP
MTHFR	MTOR	MUTYH	MYB	MYBL1	MYC
MYCL (MYCL1)	MYCN	MYD88	MYH9	NAT1	NBN
NCOR1	NCOR2	NF1	NF2	NFE2L2	NFKBIA
NKX2-1	NOTCH1	NOTCH2	NOTCH3	NPM1	NQO1
NRAS	NRG1	NSD1	NTHL1	NTRK1	NTRK2
NTRK3	NUTM1	OLIG2	OTX2	PAK3	PALB2
PALLD	PARP1	PARP2	PATZ1	PAX5	PBRM1
PDCD1 (PD1)	PDCD1LG2 (PD-L2)	PDE11A	PDGFRA	PDGFRB	PDK1
PGR	PHLDB1	PHOX2B	PIK3C3	PIK3CA	PIK3CD

PIK3R1	PIK3R2	PKHD1	PLAG1	PLCB4	PLK1
PMS1	PMS2	POLD1	POLD3	POLE	POLH
POT1	PPARD	PPM1D	PPP2R1A	PPP2R2A	PRDM1
PRDM6	PREX2	PRF1	PRKACA	PRKAR1A	PRKCI
PRKDC	PRKN (PARK2)	PRSS1	PRSS3	PTCH1	PTCH2
PTEN	PTK2	PTPN11	PTPN13	PTPRD	PTPRZ1
QKI	RAC1	RAC3	RAD50	RAD51	RAD51B
RAD51C	RAD51D	RAD54L	RAF1	RARA	RARG
RASGEF1A	RB1	RECQL4	RELA	RELN	RET
RHBDF2	RHOA	RICTOR	RNF43	ROS1	RPTOR
RRAS2	RRM1	RTEL1	RUNX1	RUNX1T1	SBDS
SDC4	SDHA	SDHB	SDHC	SDHD	SEPTIN9 (SEPT9)
SETBP1	SETD2	SF3B1	SGK1	SKP2	SLC34A2
SLC3A2	SMAD2	SMAD3	SMAD4	SMAD7	SMARCA2
SMARCA4	SMARCB1	SMARCC2	SMARCD2	SMO	SNCAIP
SOCS1	SOS1	SOX2	SPOP	SPRED1	SPRY4
SRC	SRSF2	SRY	STAG2	STAT3	STK11
STMN1	SUFU	TACC3	TAP1	TAP2	TCF12
TCF4	TEK	TEKT4	TERC	TERT	TET2

TGFBR2	THADA	TMEM127	TMPRSS2	TNFAIP3	TNFRSF11A
TNFRSF14	TNFRSF19	TNFSF11	TOP1	TOP2A	TP53
TP63	TPMT	TSC1	TSC2	TSHR	TTF1
TUBB3	TYMS	U2AF1	UGT1A1	VAMP2	VEGFA
VHL	WAS	WRN	WT1	XPA	XPC
XRCC1	XRCC2	YAP1	ZIC1	ZMYM3	ZNF217
ZNF703					

Supplementary Table 2. Clinicopathological characteristics of pediatric PDC cases (n=7)

Case No.	Age/Sex	Presenting symptoms	Location	Surgical type (resection extent), and adjuvant treatment	Follow-up duration	Vital status
1	16/F	Headache accompanied by binocular adduction	Clivus, sellar region, suprasellar region, and sphenoid sinus	Endoscopy (incomplete); adjuvant RT (proton). Tumor progression in the 11 Mo after first surgery; then second surgery.	17 Mo	Died
2	3/M	Neck pain	Clivus	Endoscopy (incomplete); no adjuvant treatment.	3 Mo	Died
3	3/F	Right upper extremity weakness	Clivus	Craniotomy (incomplete); no adjuvant treatment.	2 Mo	Died
4	6/M	Neck discomfort and left eye strabismus	Clivus	Craniotomy (incomplete); no adjuvant treatment.	2 Mo	Died
5	3/F	Right blepharoptosis and diplopia	Clivus to C2 vertebrae	Craniotomy (incomplete); no adjuvant treatment.	3 Mo	Died
6	3/F	Headache, nausea and vomiting	Clivus	Craniotomy (incomplete); no adjuvant treatment.	1 Mo	Died
7	8/M	Limited neck mobility, intermittent coughing when drinking water, and unstable walking	Clivus and right-side saddle	Craniotomy (incomplete); adjuvant RT.	22 Mo	Alive with metastasis to spinal cord and lung

M: male; F: female; RT: radiation therapy; Mo: month.

Supplementary Table 3. Clinicopathological features of 28 pediatric CC

Case No.	Age/Sex	Symptoms	Location	Surgical type (resection extent), adjuvant treatment
1	6/M	Blurred vision and binocular adduction	Clivus and petrous ridge	Craniotomy (complete), NA.
2	9/F	Limited neck mobility and left limb weakness	Clivus and C1 vertebrae	Craniotomy (complete), Tumor progression in the 12 mo after surgery, then second surgery and adjuvant RT.
3	3/M	Unstable walking	C1-C3 vertebrae	Craniotomy (incomplete), No adjuvant treatment.
4	11/M	Decreased binocular vision	Clivus and sellar region	Endoscopy (incomplete), No adjuvant treatment. Tumor progression in the 24 mo after surgery, then second surgery.
5	10/M	Headache with dizziness and nausea	Clivus and C1-C2 vertebrae	Craniotomy (incomplete), adjuvant RT. Tumor progression in the 12 mo after surgery, then second and third surgery.
6	15/M	Lumbosacral pain	S1-S3 vertebrae	Craniotomy (complete), NA.
7	11/F	Glossolalia, snore, and neck pain	Clivus	Endoscopy (incomplete), NA.
8	8/M	Neck pain and limited abilities of limbs	Clivus	Craniotomy (complete), NA.
9	15/F	Neck and shoulder pain, dysphagia, and coughing when drinking water	Clivus	Craniotomy (complete), Adjuvant RT. Tumor recurrence in the 14 mo after surgery, then second surgery.
10	18/F	Limited right eye abduction and diplopia	Clivus	Endoscopy (incomplete), adjuvant RT. Tumor progression in the 12 mo after surgery, then second surgery.

11	18/M	Double Vision	Clivus	Endoscopy (complete), NA.
12	13/F	Headache and dizziness	Sellar region	Endoscopy (complete), No adjuvant treatment.
13	7/F	Hypoevolutism	Clivus	Endoscopy (complete), NA.
14	14/M	Hypoesthesia in both upper extremities and malaise	C3-C7 vertebrae	Craniotomy (incomplete), adjuvant RT.
15	16/M	Headache with occasionally nausea	Clivus	Craniotomy (complete), no adjuvant treatment.
16	2/M	Snore and dysphagia	Clivus and C1 vertebrae	Craniotomy (incomplete), no adjuvant treatment. Tumor progression in the 9 mo after surgery, then second surgery.
17	4/F	Dizziness, hemiplegia, and anhelation	Clivus and C1-C2 vertebrae	Craniotomy (incomplete), no adjuvant treatment. Tumor progression in the 5 mo after surgery, then second surgery and RT (proton).
18	5/M	Snore	Clivus and C1 vertebrae	Craniotomy (complete), no adjuvant treatment.
19	6/F	Glossolalia, dysphagia, headache, and nausea	Clivus	Craniotomy (incomplete), no adjuvant treatment.
20	7/M	Limb weakness and headache	Clivus and C1-C2 vertebrae	Craniotomy (complete), no adjuvant treatment. Tumor recurrence in the 12 mo after surgery, then second surgery.
21	9/F	Snore, headache, pharyngeal pain, and abdominal discomfort.	Clivus	Craniotomy (complete), no adjuvant treatment. Tumor recurrence in the 36 mo after surgery.
22	10/F	Unstable walking and left leaning	Clivus	Craniotomy (incomplete), no adjuvant treatment.
23	13/M	Headache	Clivus	Craniotomy (complete), no adjuvant treatment.

24	13/F	Headache	Clivus and sellar region	Endoscopy (incomplete), no adjuvant treatment. Tumor progression in the 6 mo after surgery, then second surgery.
25	16/F	Shortness of breath, suffocation, and right hand numbness	Clivus	Craniotomy (incomplete), NA.
26	17/F	lingual atrophy, nausea and vomiting	Clivus and C1 vertebrae	Craniotomy (incomplete), no adjuvant treatment.
27	17/F	Neck and parietal-occipital region pain	Clivus and C1-C2 vertebrae	Craniotomy (incomplete), NA. Tumor progression in 12 mos.
28	17/F	Snuffling	Clivus	Endoscopy (incomplete), tumor progression in 4 mo, then second surgery and RT.

M, Male; F, Female; RT, radiation therapy; Mo, month; NA, not available.

Supplementary Table 4. Multivariate Cox regression analysis on the clinical data

Factor	HR	L95CI	H95CI	*P* value
Tumor type (PDC vs. CC)	63.80	3.46	1177.09	0.0052
age	1.03	0.85	1.26	0.74
Tumor location	>0.001	0	Inf	0.99
Resection extent	0.58	0.05	6.66	0.66
Adjuvant RT	3.03	0.30	30.17	0.34

HR: Hazard Ratio; L95CI: lower 95% confidence interval; H95CI: higher 95% confidence interval. RT: radiotherapy.

Supplementary Table 5. Differential expression patterns of SHH pathway members in seven pediatric PDC and 25 pediatric CC samples

	**PDC** (N=7)	**CC** (N=25)	***P* value**
SHH			**>0.001**
-	0	0	
+	0	0	
++	3	1	
+++	4	24	
PTCH1			**>0.001**
-	2	0	
+	2	0	
++	3	5	
+++	0	20	
SMO			**0.047**
-	0	0	
+	1	0	
++	3	3	
+++	3	22	
GLI1			**1**
-	1	0	
+	0	0	
++	5	5	
+++	1	20	

Levels of IHC staining, -: 0 - 1, 1+: 2 - 3, 2+: 4 - 8, 3+: 9 - 12; *: *P* values were derived from Mann-Whitney U test.

Supplementary Table 6. Per sample QC metrics and calibrated score for classification

Sample ID	Final Pred Class	Calibrated Score	Deta score	Median log2_Meth*	Median log2_uMeth*	detP PassPercent*
PDC_1	CHORD (DD)	0.777	0.707	11.905	11.649	99.893
PDC_2	CHORD (DD)	1.000	1.000	12.370	11.949	99.946
PDC_3	CHORD (DD)	1.000	1.000	11.823	11.483	99.929
PDC_4	CHORD (DD)	0.999	0.998	12.343	11.790	99.942
PDC_5	CHORD (DD)	0.999	0.999	11.404	11.157	99.887
PDC_6	CHORD (DD)	0.994	0.991	12.478	12.133	99.942
PDC_7	CHORD (DD)	0.235	0.084	12.081	12.054	99.970

*QC Metrics: 1) Median_log2_Meth/Median_log2_uMeth: the log2 median intensity of methylated signal, and the log2 median intensity of unmethylated signal. A value > 8 indicates a pass.; 2) detP_PassPercent: the proportion of CpG probes with a detection p-value below 0.05. A value > 0.95 indicates a pass.

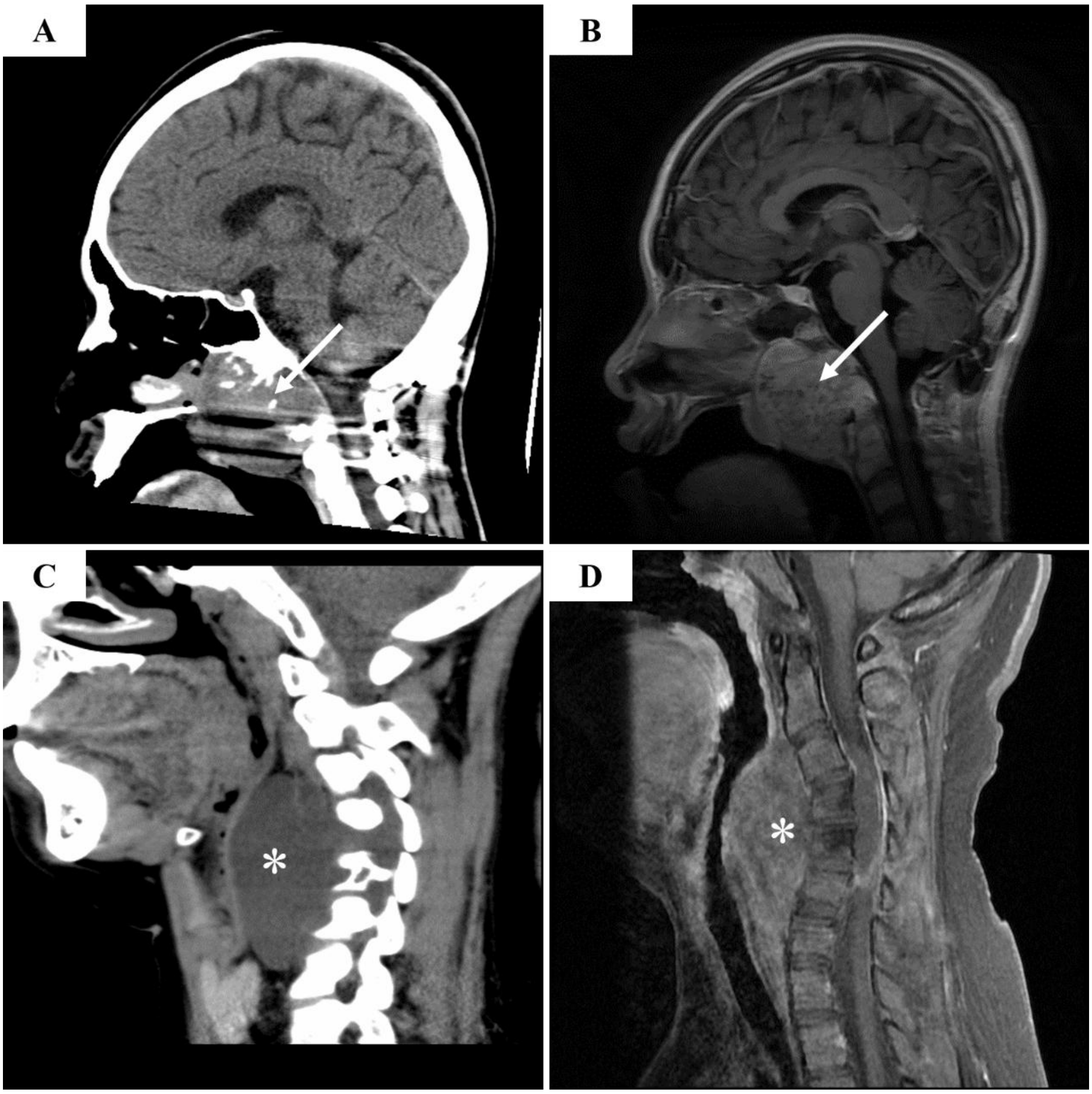

Supplementary Figure 1. Representative images of pediatric CCs.

Tumors were located at skull base (A and B, CC_28) or cervical vertebrae (C and D, CC_14). CT image shows a large clump with slight low-density on the ventral clivus and within the pharyngeal cavity (A), and the post-contrast T1-weighted image shows that the clump is unevenly enhanced (B) (white arrow indicates the tumor). CT image shows an irregular cystic mass with low density on the right side of the anterior vertebrae of C3 to C5 (C), which had a slight contrast enhancement on the post-contrast T1-weighted image (D). * in panels C and D indicates the tumor region.

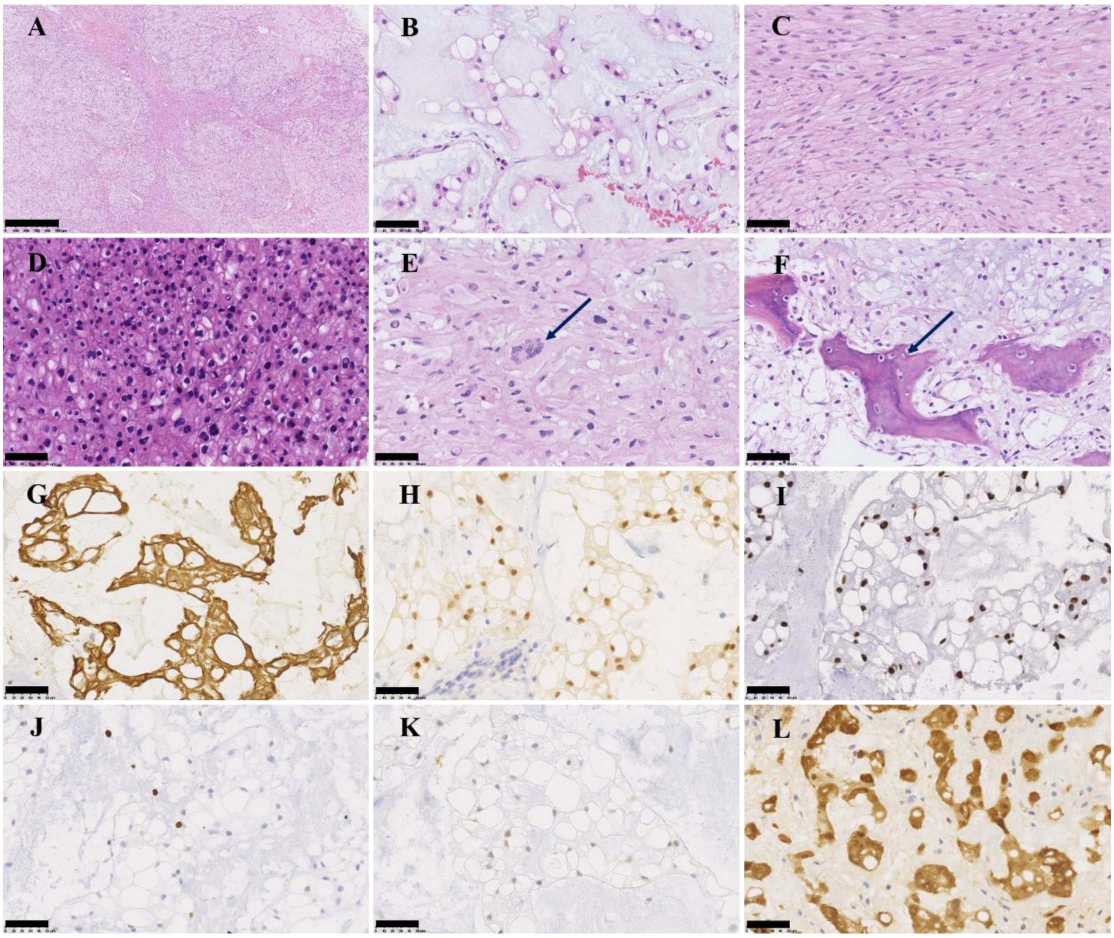

Supplementary Figure 2. Representative images of histopathology and immunophenotype of pediatric CCs.

Representative HE staining images show lobulated architecture (A), cords of tumor cells with abundant clear to eosinophilic or bubbly cytoplasm enmeshed in myxoid stroma (B), spindle tumor cells (C), high-density proliferative and atypical tumor cells (D), multinucleated tumor cells (E, indicated by black arrow), and invasion to the surrounding bone (F, residual bone indicated by the arrow). Representative immunohistostaining images show diffuse AE1/AE3 staining (G), nuclear brachyury staining (H), no loss of SMARCB1(INI1) expression (I), Ki-67 index at about 1% (J), no overexpression of P53 (K), and diffuse S-100 staining (L). The scale bar in image A measures 500 μm, the bars in all other images are scaled at 50 μm.

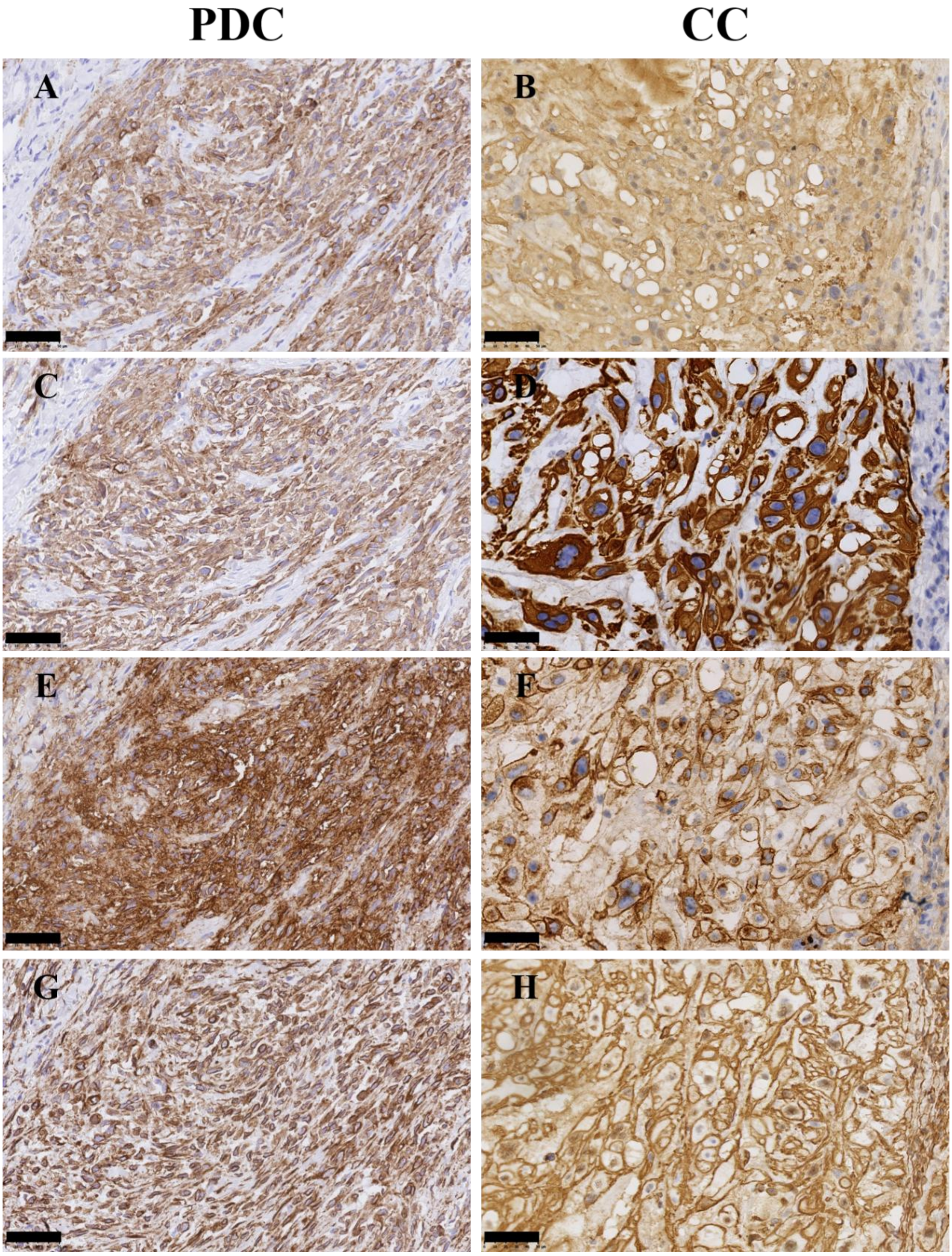

Supplementary Figure 3. Expression pattern of CK8, CK18, EMA, and vimentin in pediatric chordomas.

Representative staining images of CK8 (A, B), CK18 (C, D), EMA (E, F), and vimentin (G, H) in pediatric PDC (PDC_4, left) and CC (CC_8, right) samples are shown. Stainings were performed in consecutive sections. Scale bar: 50 µm.

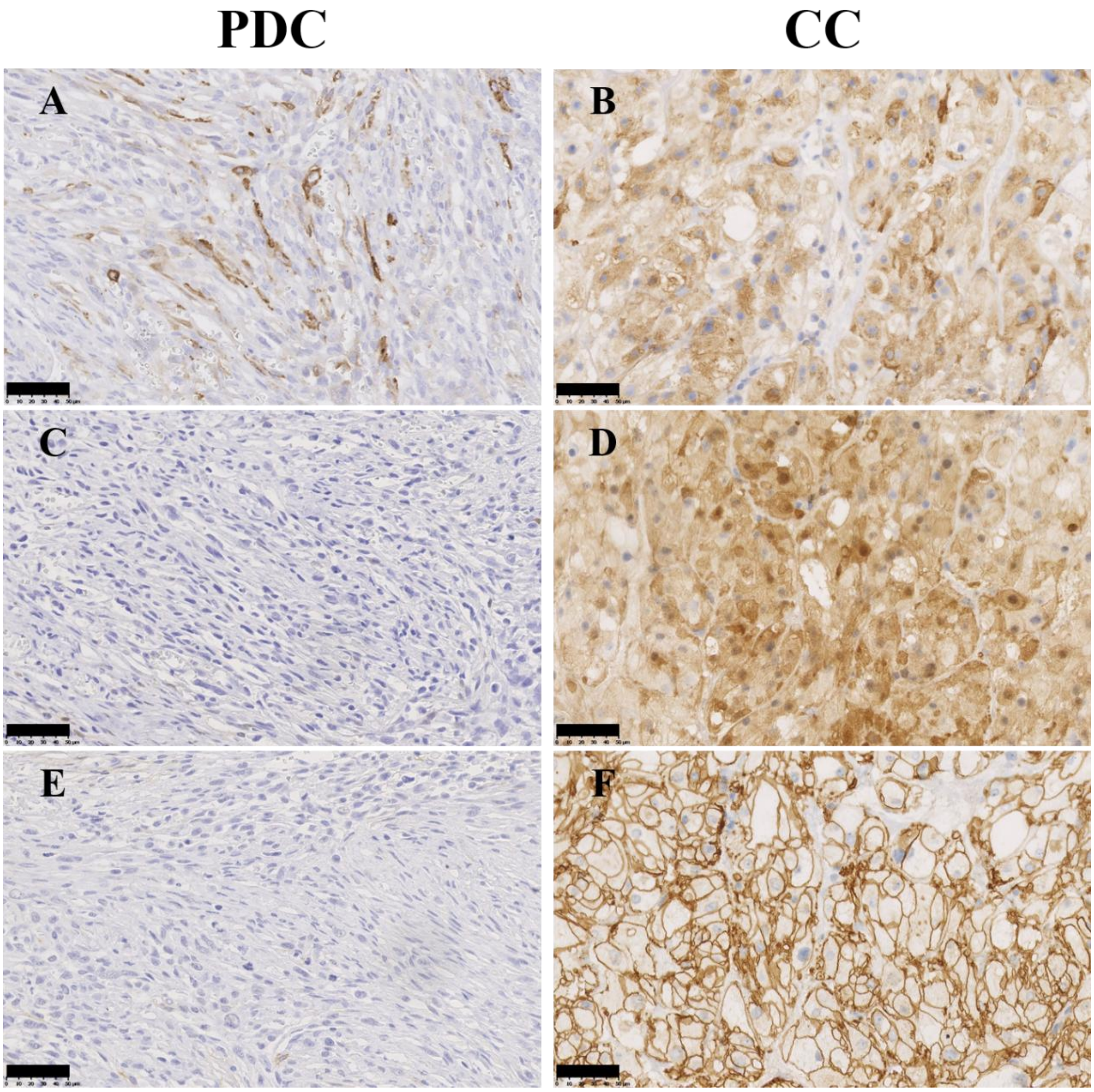

Supplementary Figure 4. Expression pattern of neural markers in pediatric chordomas.

Staining images of Syn (A, B), S-100 (C, D), and CD56 (E, F) in representative pediatric PDC (PDC_6) and CC (CC_8) samples are shown. While only focal Syn staining was detected in pediatric PDC, diffuse positive stainings of Syn, S-100, and CD56 were observed in CC. Scale bar, 50 μm.

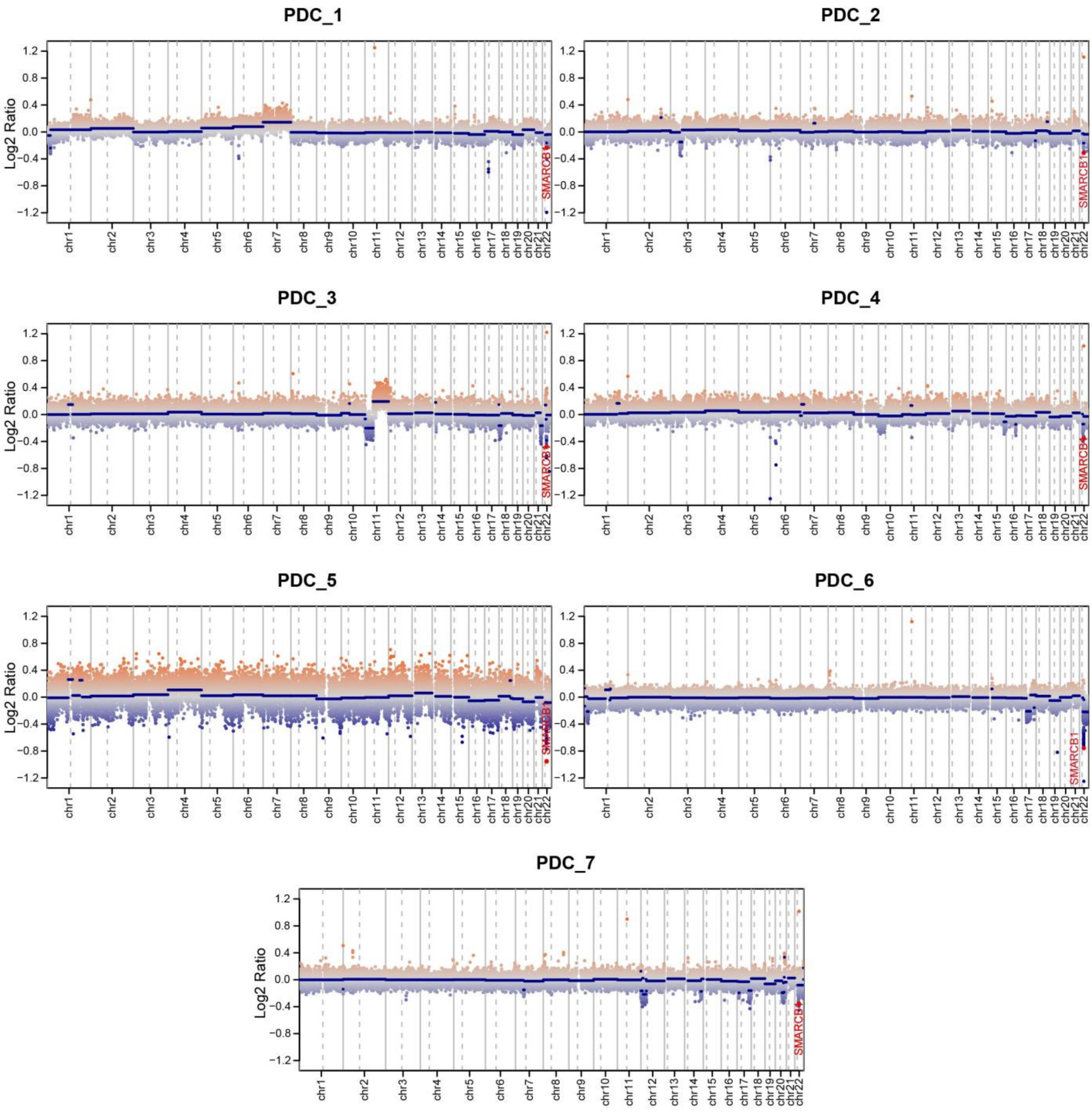

Supplementary Figure 5: Case-level methylation array-based CNV plots of seven PDC samples. The *SMARCB1* locus was annotated in each plot.

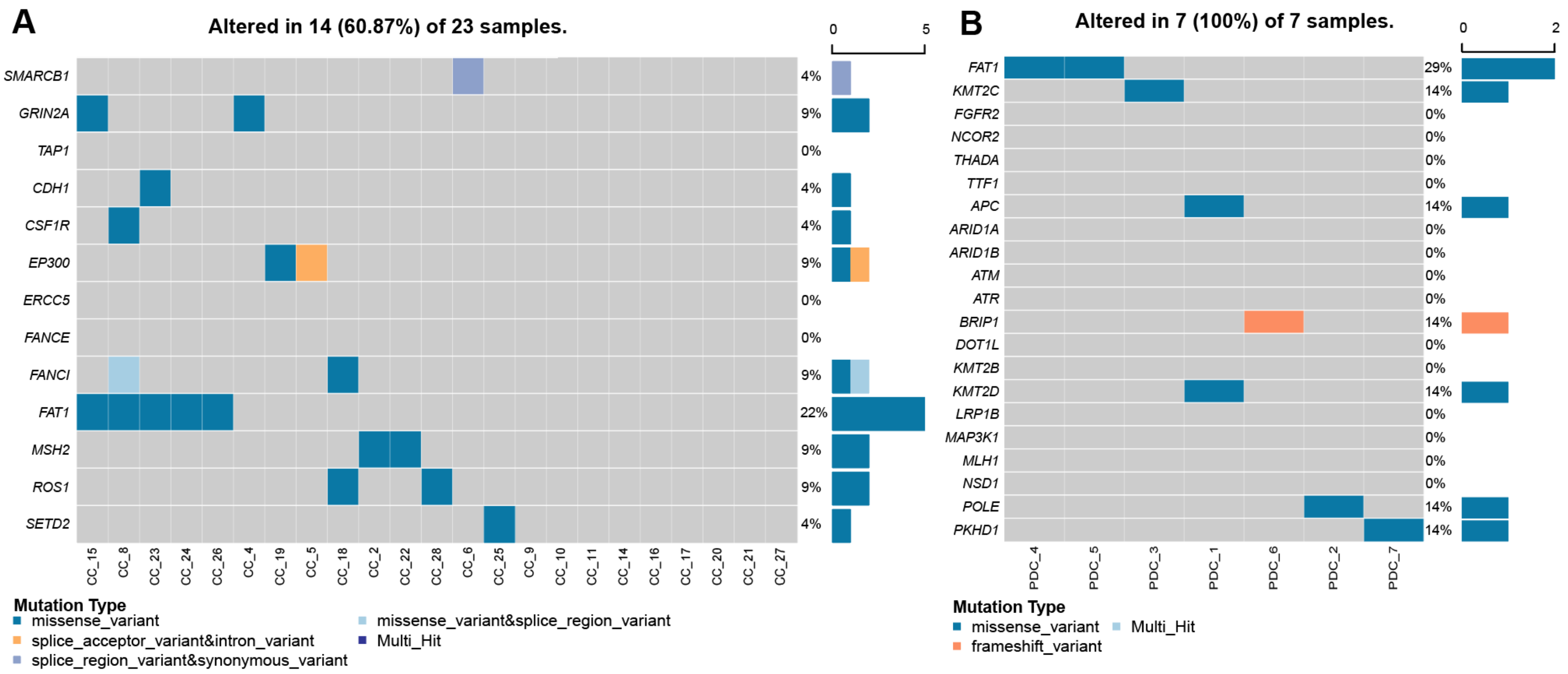

Supplementary Figure 6: Cross reference of recurrent genetic alterations between pediatric CC and PDC samples.

(A) Occurrence of PDC-enriched genetic alterations in CC samples.

(B) Occurrence of CC-enriched genetic alterations in PDC samples.
